# Old Age Bipolar Disorder—Epidemiology, Aetiology and Treatment

**DOI:** 10.3390/medicina57060587

**Published:** 2021-06-08

**Authors:** Ivan Arnold, Julia Dehning, Anna Grunze, Armand Hausmann

**Affiliations:** 1 Helios Klinik Berlin-Buch, 13125 Berlin, Germany; ivan.arnold@gmail.com; 2 Department of Psychiatry, Psychotherapy and Psychosomatics, Medical University Innsbruck, 6020 Innsbruck, Austria; 3 Psychiatrisches Zentrum Nordbaden, 69168 Wiesloch, Germany; anna.grunze@pzn-wiesloch.de; 4 Private Practice, Wilhelm-Greil-Straße 5, 6020 Innsbruck, Austria; praxis@hausmann.tirol

**Keywords:** old age, bipolar disorder, aetiology, mania, bipolar depression, mixed state

## Abstract

Data regarding older age bipolar disorder (OABD) are sparse. Two major groups are classified as patients with first occurrence of mania in old age, the so called “late onset” patients (LOBD), and the elder patients with a long-standing clinical history, the so called “early onset” patients (EOBD). The aim of the present literature review is to provide more information on specific issues concerning OABD, such as epidemiology, aetiology and treatments outcomes. We conducted a Medline literature search from 1970–2021 using the MeSH terms “bipolar disorder” and “aged” or “geriatric” or “elderly”. The additional literature was retrieved by examining cross references and by a hand search in textbooks. With sparse data on the treatment of OABD, current guidelines concluded that first-line treatment of OABD should be similar to that for working-age bipolar disorder, with specific attention to side effects, somatic comorbidities and specific risks of OABD. With constant monitoring and awareness of the possible toxic drug interactions, lithium is a safe drug for OABD patients, both in mania and maintenance. Lamotrigine and lurasidone could be considered in bipolar depression. Mood stabilizers, rather than second generation antipsychotics, are the treatment of choice for maintenance. If medication fails, electroconvulsive therapy is recommended for mania, mixed states and depression, and can also be offered for continuation and maintenance treatment. Preliminary results also support a role of psychotherapy and psychosocial interventions in old age BD. The recommended treatments for OABD include lithium and antiepileptics such as valproic acid and lamotrigine, and lurasidone for bipolar depression, although the evidence is still weak. Combined psychosocial and pharmacological treatments also appear to be a treatment of choice for OABD. More research is needed on the optimal pharmacological and psychosocial approaches to OABD, as well as their combination and ranking in an evidence-based therapy algorithm.

## 1. Introduction: Epidemiology of Bipolar Disorder in Old Age Patients

The elderly represents the fastest growing group of the population. The share of those >60 years of age has duplicated since 1980. In developed countries, the percentage of those >80 years of age will quadruple by 2050. It is fair to assume that the portion of old age patients suffering from bipolar disorder will grow in a similar manner.

Data regarding geriatric mania or bipolar disorder (BD) are sparse. Previously conducted large epidemiologic studies in BD focussed on working age adults or adolescents, e.g., [1]. Historically, there was the general notion that the prevalence of mania in the elderly decreases, making it a less important topic for research [2,3,4]. The point prevalence of bipolar disorder in older patients was assumed to be considerably less than the approximate 1% found in the general population.

However, Angst and colleagues [5] described a rate of diagnostic change from a unipolar to a bipolar I diagnosis of 1% and of 0.5% per year to bipolar II. This would result in an increase in older age bipolar disorder (OABD) at the expense of unipolar depression. A record analysis of 35,000 community patients suggests that the prevalence of bipolar disorder in older patients differs only marginally from the one in younger patients [6]. This is in line with more recent epidemiological studies that report a proportion of 0.5–1% of old age bipolar I and II patients [7,8]. Even higher numbers have been observed in special settings, e.g., a prevalence of 3–10% in nursing homes [9,10]. Dols and colleagues [11] report a 6% prevalence of manic episodes in elderly psychiatric inpatients, with 44% having late onset mania. Elderly patients (≥60 years) represent approximately 25% of the bipolar population [12], and approximately 70% of the elderly bipolar patients are female [10]. Summarizing the different studies, 5–10% of patients were ≥50 years of age when they experienced their first manic episode, constituting the subgroup of late onset bipolar disorder (LOBD) [7,10,13,14]. A second subgroup consists of elder patients with a long standing clinical history of BD, the so called “early onset” patients (EOBD).

Only few studies addressed the age of BD onset in relation to vulnerability, symptoms and prospective course. Bellevier and colleagues [15,16,17] divided patients into the following subgroups according to age at primary manifestation of BD: early, middle and late, with a mean age at onset of 17, 27 and 46 years, respectively. The groups appeared to be distinct and, within the respective group, homogeneous in clinical symptomatology and genetic vulnerability factors. In line with this, Azorin and colleagues also described distinct phenotypes in BD patients with early, middle and late onset [18].

The purpose of this educational literature review is to summarize the- still sparse-knowledge of OABD and its epidemiology, aetiology and treatments outcomes.

## 2. Methods of Literature Search

In order to gain more detailed information on OABD patients and OABD’s subgroups LOBD and EOBD, we conducted a literature review by conducting a Medline search on 3 March 2021, using, as a first step, the MeSH terms “bipolar disorder” and “aged” or “geriatric” or “elderly”. Results were further categorized and filtered by adding the following additional search terms: “etiology” or “aetiology”, “treatment”, “randomized”, “mania” or “manic”, “depression” or “depressive”, and “mixed”. The additional literature was retrieved by examining cross references and by a hand search in textbooks.

## 3. Aetiology of Bipolar Disorder in the Elderly

There is a broad consensus that OABD patients constitute a heterogenous population. Two major groups have been distinguished, LOBD and EOBD.

The dividing line between OABD and adult-age BD seems to be fluctuating, but ≥60 years of age appears to be the consensual cut-off [10,19]. The taskforce of the International Society for Bipolar Disorder (ISBD) proposes a cut-off of ≥50 years, given the reduced life expectancy in BD and to avoid studying only “the healthy cohort who survive into what our society generally considers elderly age (60+ and beyond)”. Similarly, there is some vagueness about the cut-off for what to consider as EOBD and as LOBD within the OABD group, the usually proposed ≥50 years of age might be too late, as brain-morphological changes due to neuroprogression start much earlier in life. Here, the ISBD taskforce advocates ≥40 years as a cut-off between EOBD and LOBD [19].

EOBD and LOBD appear to be distinct forms of BD [20]. LOBD patients have been reported to present more often with bipolar II disorder than EOBD patients [21]. EOBD is associated with a highly positive family history [22], whereas LOBD is frequently associated with neurological diseases, cognitive decline or other somatic conditions [23,24,25,26]. For example, in a retrospective study of 50 patients with mania who were older than 65 years, it was the first manic episode for 28% of the patients and 71% had a comorbid neurological disorder [27]. However, the data on organic factors are inconsistent. Almeida and colleges [28] pointed out that only a small proportion of old age bipolar patients have a detectable organic substrate. The authors assumed that differences between the two groups are rather due to the different duration of illness and its progression, and the contribution of organic conditions in the LOBD patients has less clinical relevance. The fact that only few LOBD patients are diagnosed with organic affective disorder—in the study of Almeida and colleagues, 2.8%—might, in part, also be explained by the fact that neurological symptoms are often subtle, and, with an obvious psychiatric symptomatology, the majority of patients might not undergo in-depth somatic diagnostics. The Diagnostic and Statistical Manual, 5th edition (DSM-5) [29] circumvents this diagnostic dilemma by also allowing a bipolar diagnosis in the presence of a potential organic origin (“bipolar and related disorders due to another medical condition”, 293.82). This approach may be clinically useful but is surely contra-productive to further aetiological research.

Apparently, there is a significant contribution of genetic risk factors in EOBD, whereas the significance of these in LOBD remains less clear. In LOBD, sensitivity to adverse drug effects, including drugs for somatic conditions, somatic disorders, e.g., inflammatory and neoplastic processes, stroke and head injuries play a considerable role [30,31], resulting in the concept of secondary mania [32]. The definition of secondary mania involves the full manifestation of a manic episode fulfilling categorial diagnostic criteria in the presence of a pharmacological, metabolic or somatic cause [33].

Studies report that 17–43% of the elderly patients with mania show symptoms of cerebral disorders [34,35,36,37,38]. Subramaniam and colleagues compared EOBD patients to LOBD patients regarding vascular risk factors, reporting that LOBD patients showed a significant higher load of risk factors [39]. The bidirectional connection between cerebrovascular diseases and unipolar depression is well proven, not only that cerebrovascular disease promotes depression, but also that later life depression is a risk factor for the development of cerebrovascular disease and dementia in general [40,41]. In BD, Kessing and Andersen [42] found that the risk of developing dementia increased with each new affective episode. About 19% of OABD patients develop dementia, almost triple the count observed in age-matched controls (7%) [43]. Most of these BD patients suffer from Alzheimer’s disease, followed by vascular dementia. LOBD patients may be at a higher risk of dementia than EOBD patients, as euthymic LOBD patients already show more cognitive impairment compared to euthymic EOBD patients. Specifically, LOBD patients show poorer performance in verbal memory and executive function tasks [26]. In addition, semantic fluency is more impaired in LOBD than EOBD patients [44].

### 3.1. Differences between OABD and BD in Working-Age Adults

The research thus far supplies inconclusive data regarding whether OABD has a better, equal or worse course and prognosis than working-age BD. The polarity of BD appears to shift with older age, with an increase in the amount of time experiencing depression, and consecutively less time spent in manic or mixed states [45].The frequency of psychotic and mixed features appears similar in OABD and working-age BD [10,46]. In the short term, the response to acute treatment and recovery rates in OABD patients appear similar to that of younger patients [47,48], but, in general and independent from age group, there is good evidence of a progressive and deteriorating course over a life span with increasing sensitization leading to more relapses after every mood episode [49,50,51]. In line with this, a potentially higher vulnerability to relapse or recurrences of OABD was also described in a large naturalistic study [48]. In addition, several studies have found a shrinking duration of the inter-episode intervals of successive episodes [52,53,54]. Contrasting these findings, a prospective follow-up of a cohort of OABD patients and a comparison to a cohort of working-age BD patients revealed only subtle differences in the long-term course. The median follow-up was 5 years. OABD patients (61.6 ± 8.3 years) spent 15, 6 and 3% of their follow-up time with depressive, manic and mixed symptoms, respectively, and experienced 4.2 ± 2.6 episodes year. No significant differences between OABD and BD in working-age patients regarding episode density or mood instability emerged in the multivariate analysis. Only a higher subsyndromal manic symptom burden was observed in OABD, impacting on functional outcomes [55]. The rates of hospitalizations seem to decrease in OABD patients, which is probably because the severity of subsequent episodes attenuates [56], and suicide rates go down, which is probably because elderly patients represent, to some degree, a selected survival cohort [10,37,57].

### 3.2. OABD with Different Symptomatology in EOBD vs. LOBD

Due to the onset of the disorder in early life, EOBD patients usually experience more severe and atypical symptoms in their first manic episode [58], as well as higher rates of psychotic symptoms and rapid cycling [59,60]. LOBD mania is usually characterized by fewer and rather milder manic symptoms compared to EOBD [61]. On the other hand, LOBD patients show more frequently irritable behaviours, develop more often treatment resistance and have higher mortality rates [62].

Regarding suicidality, the data are inconsistent. Both higher [60] and lower [18] suicidality rates have been reported for EOBD vs. LOBD

In summary, it appears that the acute—especially manic—symptomatology is more pronounced in EOBD, whereas maintaining cognitive functioning is more difficult in LOBD patients [26].

The already cited study conducted by Azorin and colleagues also described distinct phenotypes in BD patients with early, middle and late onset [18]. Patients in the early onset subgroup were more often single young males exhibiting severe mania with psychotic features, a subcontinuous course of illness with substance use and panic comorbidity, more suicide attempts and temperamental components sharing hypomanic features. The patients with late onset showed a less severe picture with more depressive temperamental components, alcohol use and comorbid general medical conditions. These differences in illness characteristics may also translate into differences in educational achievements, stable social relationships, social adjustment and support, and resources to cope with the disease, which, in turn, are likely to impact on the long-term course and prognosis; however, this hypothesis still needs to be backed up by data.

## 4. Treatment of OABD

### 4.1. Psychopharmacological Treatment

Medication studies in BD usually address working-age patients and exclude elderly patients, who are commonly ≥65 or 70 years old. Up to now, no large-scale, randomised and placebo-controlled study on the efficacy and tolerability of medication exclusively in OABD patients has been published. None of all of the cited studies distinguished between EOBD and LOBD. Thus, it remains unclear, and subject to further studies, whether there is a difference among both groups in terms of response to pharmacotherapy and adverse drug effects.

A Medline search found two randomized, double-blind comparator studies in acute mania (comparing lithium to valproate [63] and lithium to memantine [64]). Furthermore, we identified a post-hoc analysis [65] of pooled data from two quetiapine monotherapy clinical trials in patients aged ≥55 years, and one study each in bipolar depression (a post-hoc analysis of two placebo-controlled, 6-week, randomized, double-blind studies with lurasidone [66]) and maintenance (a post-hoc analysis of two double-blind maintenance studies comparing lamotrigine, lithium and a placebo [12]). Despite the sparse evidence, some guidance on the management and treatment of BD in old age has been compiled, e.g., [12,67,68,69,70]. In the absence of contradicting evidence, current guidelines concluded that first-line treatment for old age BD should be similar to that for working-age BD, with specific attention to vulnerability to side effects, somatic comorbidities and specific risks in elderly patients, e.g., usage of antipsychotics in cerebrovascular disease [71].

### 4.2. Treatment of Mania and Hypomania

The evidence for the treatment of acute mania with lithium is based on a randomized, controlled comparator study against valproate [63], reporting both medications as equally effective. Sanderson and colleagues compared the length of stay and the symptom improvement in elderly BD inpatients treated with lithium, valproic acid or carbamazepine as a monotherapy and found no significant differences across the groups [72]. A retrospective study conducted by Chen and colleagues also found a comparable antimanic effect of lithium and valproic acid [73]. Open naturalistic data from the STEP-BD study also support the use of lithium; 79% of elderly BD patients achieved remission after 8 weeks and 42% of these had lithium prescribed as a monotherapy [74]. Open studies also suggested the acute antimanic efficacy of valproic acid alone or in combination with lithium [75,76,77,78,79,80,81,82]. A study in 70 elderly bipolar patients in acute mania compared memantine vs. a placebo add-on to valproic acid and reported a significant advantage in the YMRS score’s reduction for this combination, but there was no difference in improving cognition [64]. Post hoc analysis of pooled data from two quetiapine monotherapy clinical trials in patients aged ≥55 years also favoured quetiapine over a placebo [12]. A small open pilot trial was also supportive for the use of asenapine in geriatric mania [83], and case reports suggest the efficacy of carbamazepine [72], gabapentin [84] and clozapine [85].

### 4.3. Treatment of Bipolar Depression

A 12-week, open-label trial of a lamotrigine augmentation found a significant antidepressive effect on OABD [86], but, again, confirmative controlled studies are still missing.

A post-hoc analysis supports the efficacy of lurasidone in acute bipolar depression as a monotherapy but not as an add-on treatment in refractory patients [66]. The data from two placebo-controlled, 6-week, randomized, double-blind studies were compiled, a monotherapy [87] and an add-on study to lithium or valproic acid [88]. Lurasidone was found to be superior to a placebo in the monotherapy study, specifically in the OABD concerning the change in the MADRS score from baseline to endpoint. In the second study, there was no difference between the adjunctive lurasidone and a placebo.

We could not identify any controlled study of antidepressants in the elderly with acute bipolar depression. As far as the risk of manic switches is concerned, they may be safer in OABD patients than in younger patients as the natural odds of a manic recurrence also decreases with age [45]. Furthermore, at least serotonin-reuptake inhibitors (SSRIs) are usually well tolerated and safe, and might have additional benefits for health, e.g., a reduction in mortality from myocardial infarction [89]

More recent, the importance of inflammation, oxidative stress and mitochondrial dysfunction in the progression of BD has been recognized [90]. A small open add-on study with the mitochondrial modulator and antioxidative substance Coenzyme Q10 in geriatric BD patients with depression showed a significant reduction in MADRS scores over four weeks [91]; however, confirmative trials are still missing.

When medication fails, electroconvulsive therapy (ECT) is a treatment of choice to treat both mania and depression [92], and can also be used in continuation treatment [93]. Studies in older patients with unipolar depression have demonstrated that not only mood but also cognitive symptoms may improve with ECT [94,95,96]. Greenberg and Kellner [97] proposed that, similar to working-age patients, the use of ECT in old age BD patients may be most useful for patients with treatment refractoriness to medication, in those refusing fluids and foods, or individuals with severe suicidal thoughts. However, more rigorous research on the effectiveness and safety of ECT in the elderly with BD is needed.

### 4.4. Treatment of Mixed Episodes

Manic mixed episodes might be of less clinical importance in OABD patients as polarity appears to shift with older age, with an increase in the amount of time experiencing depression, and consecutively less time spent in manic or mixed states [45]; however, older studies report similar figures of mixed features in OABD and younger BD patients [46]. A controlled study conducted by Young and colleagues [63] comparing lithium and valproic acid also randomized 28 mixed patients; however, this sample was too small to allow for a separate analysis. Thus, it remains unclear whether the treatment of mixed states in OABD patients should differ from treatment in younger BD patients.

### 4.5. Maintenance Treatment

No controlled randomized maintenance study has been conducted exclusively on OABD. The only available reasonable evidence stems from a post-hoc analysis [12] of 98 patients aged 61.0 ± 6.0 (range 55–82) with BD-I, which had participated in two double-blind maintenance studies comparing lithium, lamotrigine and a placebo [98,99]. The primary outcome was the time to intervention for any emerging mood episode. There was no difference among the three arms in terms of the prevention of any mood episode after adjusting for an index episode. Lamotrigine was more efficacious than lithium and the placebo in the prevention of depressive episodes (*p* = 0.01), while lithium did not differ (*p* = 0.08) in comparison to placebo. Lithium, but not lamotrigine, significantly delayed the time to intervention for a manic/hypomanic/mixed episode in comparison to a placebo (*p* = 0.034). However, when results were adjusted for an index episode, the differences became non-significant.

In summary, the results of this study support the efficacy of lamotrigine in the prevention of depression but not mania, whereas the effect of lithium on the prevention of either mania or depression in OABD patients was not significant. Nevertheless, lithium is considered as the first line medication for OABD maintenance treatment, recommended both for the prevention of depression and mania [100].

The evidence for the use of antipsychotic drugs in the maintenance treatment of OABD is still limited [101]. Tournier and colleagues [102] investigated the ates of treatment discontinuation, switch, adjunctive medication, hospitalization, suicide attempt and death over a 1-year period in a historical BD cohort using the French national healthcare database. The patients were treated with either mood stabilizers (lithium, valproic acid, carbamazepine and lamotrigine), second generation antipsychotics (SGA) (risperidone, aripiprazole, quetiapine and olanzapine) or a combination of the two classes. Looking into the subgroup of patients ≥65 years of age (*n* = 3862), treatment failure was higher in those receiving SGAs than mood stabilizers, and early discontinuation, psychiatric hospitalizations and death occurred more frequently in patients who were prescribed SGAs. Mortality was particularly high in SGA-treated elderly patients, either as a monotherapy or in combination with mood stabilizers [102]. The capability of several atypical antipsychotics to facilitate metabolic syndrome [103,104] may have a detrimental impact on mortality rates. Thus, and in the absence of convincing evidence for the use of SGAs in elderly BD patients, mood stabilizers rather than SGAs appear to be the treatment of choice for OABD.

However, also with the use of mood stabilizers, there are important safety aspects that need to be considered for OABD. The impact of lithium on renal, thyroid and parathyroid function is well known, and especially a diminishing renal function in the elderly may constitute a problem. However, valproic acid has also shown an association with renal failure [105]. Doses of lamotrigine need to be adapted with ceasing renal function. For a more detailed review on the side effects and safety profile of mood stabilizers and SGAs in the elderly, we refer the reader to the comprehensive literature [19,106,107]. Furthermore, co-medication with drugs for somatic disorders is frequent in old age. The administration of lithium together with angiotensin converting enzyme (ACE) inhibitors, calcium antagonists, thiazide diuretics and loop diuretics as well as COX-2 inhibitors and non-steroidal anti-inflammatory drugs can increase lithium serum levels and cause toxic symptoms [108]. The drug interactions between valproic acid and aspirin, digitoxin, phenytoin and lamotrigine are well documented and need to be kept in mind [109].

### 4.6. The Role of Psychotherapy in OABD

The psychotherapeutic approaches to BD with good evidence include cognitive behavioural therapy, psychoeducation, family-focused therapy and interpersonal and social rhythms therapy [110]. In OABD, the evidence for the usefulness of psychotherapies in the management of bipolar disorder is much weaker. As in working-age BD, combined psychosocial and pharmacological treatments appear to be the treatment of choice in older adults with bipolar depression (e.g., [111,112]) with similar response rates when compared to working-age BD patients. Cruz and colleagues found that non-adherence and lack of knowledge about bipolar disorder and the need for treatment was significantly worse in older BD patients [113], calling for a psychoeducational approach. Specifically for middle- and older-age adults, Depp and colleagues developed and tested a program that aimed to improve medication adherence. Their pilot study found that their psychosocial program is effective in improving not only adherence, but also depressive symptoms and quality of life as well [114]. In addition, more recent psychotherapeutic developments using technical devices might be suitable for OABD. Contrasting common beliefs, Fortuna and colleagues showed that older adults with serious mental illness and limited technical abilities are capable of using smartphone applications for self-management if they are designed appropriately [115].

## 5. Conclusions

The share of OABD will grow constantly in the next decades and further research on this neglected patient group is urgently required. There is a broad consensus that OABD patients constitute a heterogenous population, and two major groups have been distinguished, namely LOBD and EOBD. The current studies on the treatment of OABD do not distinguish between EOBD and LOBD and whether there is a difference among both groups in terms of pharmacotherapy and adverse drug effects remains subject to further investigation.

Due to a lack of data on the treatment of OABD, current guidelines concluded that first-line treatment of OABD should be similar to that for working-age BD, with specific attention to the vulnerability to side effects, somatic comorbidities and specific risks in elderly patients.

With reliable and constant monitoring, while being aware of possible toxic drug interactions, lithium is a safe drug for the treatment of manic episodes as well as for the maintenance therapy of OABD. For bipolar depression, there is some evidence for the use of lamotrigine and lurasidone. SSRIs are well tolerated, safe and might have additional benefits for health; however, no controlled trials have been conducted thus far on the use of antidepressants for OABD. No specific recommendation can be given for mixed states in OABD. Mood stabilizers rather than SGAs appear to be the treatment of choice in terms of the maintenance therapy for OABD.

When medication fails, ECT is a treatment of choice to treat both mania and depression, and can also be used in continuation treatment.

Psychoeducation is also essential for OABD patients, as it is for young BD patients, and combined psychosocial and pharmacological treatments appear as well to be the treatment of choice in older adults with bipolar depression and for maintenance. However, more research into OABD in general is needed, and it still remains speculative whether treatment algorithms should differ between EOBD and LOBD.
